# “Green” PBX Formulations Based on High Explosives (RDX and HMX) and Water-Soluble pH-Sensitive Polymeric Binders

**DOI:** 10.3390/polym15071790

**Published:** 2023-04-04

**Authors:** Traian Rotariu, Andreea Elena Moldovan, Gabriela Toader, Aurel Diacon, Edina Rusen, Raluca Elena Ginghina, Ovidiu Iorga, Horia Răzvan Botiș, Thomas Klapötke

**Affiliations:** 1 Military Technical Academy “Ferdinand I”, 39–49 George Cosbuc Boulevard, 050141 Bucharest, Romania; 2 Faculty of Chemical Engineering and Biotechnologies, University Politehnica of Bucharest, 1–7 Gh. Polizu Street, 011061 Bucharest, Romania; 3 Research and Innovation Center for CBRN Defense and Ecology, 225 Olteniţei Ave., 041327 Bucharest, Romania; 4 Compania Națională ROMARM S.A., 5 Timișoara Boulevard, 061301 Bucharest, Romania; 5 Department of Chemistry, Ludwig-Maximilian University of Munich, Butenandtstr. 5–13, 81377 Munich, Germany

**Keywords:** plastic-bonded explosives (PBX), RDX, HMX, polymers, binders, water-soluble, pH sensitive

## Abstract

The increasingly harsher and more complex international and European environmental legislation drives the current development of “greener” energetics materials and munitions. The aerospace and defense industries rely on extensive research in the formulation and scale-up production of polymer-bonded explosives (PBX). In this context, this paper aims to present a versatile method for obtaining “green” PBX formulations based on two high explosives (hexogen (RDX) and octogen (HMX)) and acrylic acid—ethyl acrylate copolymeric binders. This study developed an innovative “eco-friendly” technology for coating the RDX and HMX crystals, allowing straightforward and safer manufacture of PBX, avoiding the use of traditional organic solvents. At the same time, these polymeric binders are soluble in water at a slightly alkaline pH and insoluble at acidic or neutral pH, thus ensuring a safer manipulation of the energetic materials during their entire life cycle and a facile recovery of the explosive in its original shape and morphology in demilitarization. The PBX formulations were characterized via specific analytical tools to evaluate the influence of their composition on the safety and performance characteristics: scanning electron microscopy (SEM), Fourier-transform infrared spectroscopy (FT-IR), alkaline pH solubility tests, differential thermal analysis (DTA), impact sensitivity test (BAM Fall Hammer Test), friction sensitivity test (BAM Friction Test), electrostatic sensitivity test (ESD), vacuum stability test, small scale shock reactivity test (SSRT), detonation velocity test. The “green” PBX formulations obtained through a simple and innovative coating method, based on the polymeric binders’ adjustable water solubility, demonstrated remarkable energetic performances and a facile recovery of the explosive crystals by the dissolution of the polymeric binder at pH 11 and 30 °C.

## 1. Introduction

In the last few years, people have become more aware of the negative effects of military actions on the environment. Consequently, all nations must implement policies to guarantee that national defense activities have a reduced environmental footprint. Therefore, safer and environmentally friendly energetic formulations should be developed to minimize environmental and human health impacts.

There is a risk for long-term air, soil, and water pollution caused by the use of energetic materials (EMs). EMs pose severe environmental risks because of their hazardous nature and toxicological characteristics [1,2,3]. In addition to acceptable safety, high energy and a controllable reaction rate are two other prerequisites for energetic materials [4].

The growing need for amenable environmental policy development had become clear, especially in the last years, when many manufacturing sites and military bases were closed, and large amounts of armament systems were demilitarized. In the last two decades, there has been increased social and legal pressure on manufacturers and users of EMs and munitions to diminish their products’ environmental impact and comply with national and international environmental regulations.

Furthermore, the necessity of developing safer ammunition and finding reliable and efficient methods for demilitarizing and disposing of ammunition [5,6] has become a challenge for researchers because this process is complex, dangerous, and expensive. In this context, developing safer and greener energetic formulations is mandatory to reduce environmental and human health effects [7].

Recycling the explosives from munitions is often seen as a way of covering the costs of disposal, as the recovered energetics can possibly be reused for civil and military applications. The literature [8,9] presents techniques such as supercritical fluid extraction, liquid ammonia extraction, and organic solvent extraction that can produce recovered material that may be acceptable for use. But the major drawback is the need to satisfy authorities regarding the consistency and safety of the recovered materials. These materials must be demonstrated to be safe in themselves and that no contaminants remain, which will prevent safe use and significantly add to the cost [8].

A densely packed composite material comprising EMs crystals (secondary high explosives) and a polymeric binder (approx. 5–10 wt.%) is known as a polymer-bonded explosive (PBX) [10]. PBXs are employed in civil and military applications when exceptionally high performance is necessary [10]. The most commonly used energetically inert binders are hydroxyl-terminated polybutadiene (HTPB), polytetrafluoroethylene (PTFE), Viton A (copolymer of vinylidene fluoride and hexafluoropropylene), styrene–butadiene rubber (SBR) or thermoplastic elastomers (TPE)—thermoplastic polyurethanes (TPU), thermoplastic polyolefins (TPO), thermoplastic polyamides (TPA), thermoplastic styrene block copolymers (TPS), and thermoplastic polyesters (TPC) [11].

Since there are fewer and fewer producers of PBX ingredients, and strong non-EU dependencies are becoming a real issue, the recovery and reuse of EMs components from PBXs are becoming more and more attractive due to their increasing production price and low availability. Conventional methods for demilitarization are based on solvent solubilization or water jet removal of EMs from the ammunition shell [12]. After removal from ammunition, the EM can be neutralized, or it can be recovered, recycled, and reused [13]. In addition, the purity and shape of the energetic materials can be altered during the EM recovery operations [13]. However, as open burning (OB) and open detonation (OD) are limited or no longer accepted in many countries, researchers have been looking to develop new demilitarization methods [14]. Often solvent solubilization can be applied to dissolve the binder and recover the explosives [15]. However, most solvents still carry risks to the health of the operators and environmental issues and will modify the morphology of the explosive crystals. The use of thermoplastic polymers as binders for explosives enables the recovery and reuse of the explosive formulation, lowering the life-cycle wastes [16]. However, this method does not allow the recovery of the explosive filler (crystals). It only facilitates the extraction of the formulation from munition by melting and reuse by casting as it is or with some ingredient additions.

The most performant binders are crosslinked polymers that serve as a three-dimensional matrix for binding the EMs components, forming a tough, elastic network structure capable of absorbing and dissipating energy from harmful stimuli [10]. Yang et al. prepared RDX, HMX, and CL-20—core–shell structured micro-energetic materials [17] via in situ polymerization of melamine–formaldehyde (MF) resins [18] on the explosive crystals. Unfortunately, when employing crosslinked binders, their dissolution is impossible, thus complicating the recovery process of EMs crystals.

Recent studies [19] indicated that coating the crystals of a high explosive (e.g., HMX) with small amounts of common polymers (polyacrylates, polyacrylonitrile, polybutadiene-styrene rubber, ethyl cellulose) can effectively absorb the mechanical stress, resulting in a much lower sensitivity to impact, considerable improvement of the friction sensitivity, and increased flowability of the resulting granular material. Moreover, acrylic polymers showed the best adhesion force over the composite surface, the best flowability, and the lowest sensitivity to friction and impact mechanical stimulus.

In our previous work [7], we developed eco-friendly binders that allowed facile recovery of an inert filler by dissolving the binders in an aqueous solution at alkaline pH without using organic solvents for the binder removal process. However, in this preliminary study [7], the coating of the explosives crystals with the polymeric binder was performed with a conventional method, using a lacquer of the polymer in an organic solvent (N, N-dimethylformamide—DMF) and drying/granulation processes similar to those involved in pyrotechnics manufacture [20]. That could be a serious drawback when considering application in large-scale manufacture due to the increased sensitivity of the explosive ingredients and the cost and environmental impact of the solvent used (DMF).

In this context, this study aimed to develop a novel “eco-friendly” coating technology for the manufacture of energetic compositions based on the adjustable water solubility (at basic pH) of the polymeric binders (copolymers of alkyl acrylate and acrylic acid) previously developed by our team [7]. Due to its chemical characteristics, the binder will also allow facile embedding and recovery of the explosive filler. The recovered explosive maintains its physical, chemical, and explosive properties, making the process more feasible for the defense industry. Another aim was to determine/evaluate some physical, chemical, and performance characteristics of the new PBX formulations. Furthermore, the recovered energetic material (HMX) will be tested, and the results obtained will be compared with the literature data.

The development of a binder with selective (pH-sensitive) water solubility is of great interest from a practical point of view. As mentioned before, typically, organic solvents are employed for the formation and destruction of the polymeric binders, increasing the costs, risk, and environmental impact [12]. By using a pH-sensitive binder, not soluble at neutral pH, the water insolubility characteristic of a typical PBX is reached, while the solubility at basic pH offers a safe and cost-effective way to coat the explosive crystals and eventually recover them in their original shape and size for reuse.

## 2. Materials and Methods

### 2.1. Materials

The following monomers were used for the synthesis of the copolymeric binder: ethyl acrylate (EtAc, 99.5%, Sigma Aldrich, St. Louis, MO, USA) and acrylic acid (AAc, 99%, Sigma Aldrich, St. Louis, MO, USA), solvent: ethyl acetate (99.5%, Sigma Aldrich, St. Louis, MO, USA). α,α′-Azoisobutyronitrile (AIBN, 98%, Sigma Aldrich, St. Louis, MO, USA), recrystallized from methanol (solvent, 99.8%, Sigma Aldrich, St. Louis, MO, USA), was used as the initiator. Firstly, the monomers, AAc and EtAc, were purified by vacuum distillation. Distilled water, NaOH, HCl standard solutions (CHIMOPAR, Bucharest, RO, USA), N,N-Dimethylformamide (DMF, 99.8%, Sigma Aldrich, St. Louis, MO, USA), were used to dissolve the polymers, and for the precipitation process. The explosive crystals used in the experiments were provided as follows: FOX-7, NTO, and HMX were procured from Dyno Nobel. RDX was synthesized at the Military Technical Academy, Bucharest, using hexamine nitration with fuming nitric acid, followed by recrystallization processes to regulate the crystal morphology. Acetone (CHIMOPAR, 99%) was used for RDX recrystallization.

### 2.2. Methods

#### 2.2.1. Synthesis of the Copolymer Binder

The acrylic acid–ethyl acrylate copolymers were obtained according to the procedure presented in Reference [7]. The monomers, the initiator, and the solvent (ethyl acetate) were added in a glass round-bottom reactor flask, the initiator representing 1 wt.% of the monomer mixture. Initially, nitrogen gas was purged for 15 min. The solution was subsequently heated and maintained at 65 °C, under vigorous stirring, for 24 h. The resulting polymers were precipitated by dropping the ethyl acetate polymeric solution in petroleum ether under stirring. The obtained polymeric particles were dried in a vacuum desiccator. The two copolymers synthesized for this study contained AAc:EtAc in a 7:3 and 5:5 molar ratio, respectively. These copolymers demonstrated excellent solubility in alkaline solutions at relatively low temperatures, as was previously shown in Reference [7]. Based on the information obtained from the solubility tests in alkaline pH, Table S1 in the Supplementary Information file, the copolymers with 7:3 (AAc:EtAc) were further chosen for PBX manufacture since they exhibited good solubility at pH = 11–13 for temperatures between 20 and 50 °C, after 24 h.

Table 1 summarizes all three types of PBX formulations developed in this study according to the below-described manufacturing methods: solvent evaporation (see Section 2.2.2) and pH-variation precipitation (see Section 2.2.3).

#### 2.2.2. pH-Sensitive Binder—PBX Formulations Obtained via the ‘Classical’ Method: Solvent Evaporation

The conventional method [21] of obtaining PBX compositions implies the dissolution of the binder in the appropriate solvent, followed by mixing this polymeric solution with the energetic material and solvent evaporation at the end of this process. Thus, to obtain a “classical” polymer/high explosive composite (HMX-AAc/EtAc in our case), the 3:7 (AAc/EtAc) copolymer was dissolved in N,N-Dimethylformamide (sample code PBX 3—Table 1). After the complete dissolution of the polymer, the energetic material was added. The mixture was allowed to dry partially for 1 h, at room temperature, by slowly mixing, and then it was granulated by being passed through a 2-mm sieve. The resulting granular composite material was dried at 50 °C in an oven. After drying, to remove very small-sized particles and large-sized conglomerates, the PBX 3 mixture was granulometrically sorted by collecting the fractions between 0.2–2 mm. The polymer content represents 10% of the total mass of the resulting PBX composite. The aspects of PBX 3 (HMX—7:3 AAc/EtAc) before and after solvent evaporation (granular, odorless, white compound) are illustrated in Figure S1c in the Supplementary Information File.

We evaluated the processability of the composite material obtained, the adhesive capacity of the polymeric binder, and the possibility of recovering the explosive crystals after binder dissolution in an alkaline solution. As such, PBX 3 pressed cylinders were obtained as further described: 2 g portions of the granular composite material PBX 3 were pressed in a 10 mm diameter steel mold, in cylindrically shaped pellets, at 1400 bar, using a hydraulic press. Thus, six cylindrical charges (Ø10 mm) containing 2 g of PBX 3 were obtained, and their performances were evaluated as described in Section 2.3 below.

#### 2.2.3. pH-Sensitive Binder—PBX Formulations Obtained via Innovative “Green” Method: Precipitation of Polymer on the Surface of the Explosive Crystals

The novel composite materials (PBXs—polymer bonded explosives) based on RDX and acrylic acid–ethyl acrylate copolymer were obtained by dispersing the RDX crystals in an acidic aqueous solution (0.5 N and 0.1 N HCl solutions), dissolving the polymer in a basic aqueous solution (0.1 N NaOH solution) at moderate temperatures (20 ÷ 30 °C, 6 h vigorous mixing), followed by the addition (dropwise) of an appropriate quantity of the basic polymeric solution to the acidic solution with the dispersed RDX crystals, under vigorous stirring (700 rpm), at lower temperatures (0 ÷ 5 °C). Due to instant pH and temperature changes, the acrylic copolymers precipitated from the alkaline solution, covering and thus stabilizing the RDX crystals. Depending on the additional frequency and stirring rates, the copolymers precipitate on the surface of the RDX crystals and generate granules more or less adherent to each other, suitable for further processing by filtration, several washing steps with an acidic solution and water, drying at room temperature for 24 h, followed by oven drying at 60 °C for 2 h.

**Figure 1:**
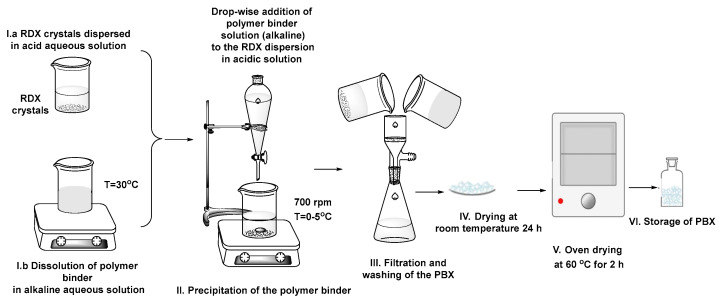
Schematic representation of the development process of PBX 1 and PBX 2 formulations. Legend: I.a—dispersing the RDX crystals in an acidic aqueous solution (0.5 N and 0.1 N HCl solutions); I.b—dissolving the copolymer binder in an alkaline aqueous solution (0.1 N NaOH solution); II—addition (dropwise) of the alkaline polymeric solution, vigorous stirring (700 rpm), 0 ÷ 5 °C; III—filtration, several washing steps with acidic solution and water; IV—drying at room temperature for 24 h; V—oven drying at 60 °C for 2 h; VI—storage of PBXs. Formulations: PBX-1—RDX suspension in 0.1 N HCl; PBX-2—RDX suspension in 0.5 N HCl; Final compositions contain 10% binder and 90% RDX.

Figure 1 illustrates the above-described manufacturing process.

#### 2.2.4. High Explosives Safe Disposal from a Cylindrical Pressed Charge (PBX 3) via Dissolution of Binder in pH = 11 at 30 °C in 48 h

For this experiment, six cylindrical charges (containing 2 g of PBX 3 (HMX-AAc/EtAc composite), 10 mm in diameter, were obtained using a hydraulic press. Subsequently, an alkaline solution with a pH of 11 was used to establish the simple recovery of the energetic material from the PBXs, at 30 °C in 48 h. Each of the cylindrical PBX 3 charges was introduced in 200 mL alkaline solution for 48 h at room temperature and 30 °C. After 48 h, the polymer was entirely dissolved, and HMX was sedimented on the bottom of the vials.

### 2.3. Characterization

The morphological analysis of the RDX or HMX crystals and the new composite granules (PBX 1, PBX 2, and PBX 3) was acquired using a Tescan Vega II LMU scanning electron microscopy (SEM) coupled with a Bruker Quantax XFlash 6/10 energy-dispersive X-ray spectroscopy (EDX) detector. The SEM-EDX analyses were performed in a high vacuum regime (0.06–0.07 Pa), using an SE detector at an acceleration voltage of 1.64 keV and magnification between 80× and 1.5k×. EDX elemental mapping and the spectrum quantification with P/B-ZAF were performed at an input count rate (ICR) of 2kcps using the Esprit software.

FT-IR spectra were obtained using a Perkin Elmer Spectrum Two (USA) with a Pike Miracle ATR modulus by collecting 32 scans, at 4 cm^−1^ resolution, from 550 to 4000 cm^−1^.

Samples weighing approximately 25 ÷ 30 mg were subjected to thermal investigations, being heated from 30 °C to 450 °C with a constant heating rate of 5 °C/min on a DTA OZM 551 Ex Differential Thermal Analysis System (Czech Republic) equipped with Meavy dedicated software.

The friction sensitivity was determined using a BAM Friction sensitivity tester, following NATO standard STANAG–4487 [22]. The friction apparatus comprises a cast steel base on which the precise friction device is mounted. It includes a fixed porcelain peg and a pivoting porcelain plate. The energetic materials were placed between the ceramic cylinder and the ceramic plate, passing one over the other while the apparatus applied various pressing forces. The friction sensitivity was determined as the minimum loading (N) that initiates 50% of the samples after 10 experiments.

The impact sensitivity test (Fall Hammer Test) was used to determine the sensitivity of explosive materials to impact stimuli from a falling drop weight in the range of impact energies between 0.25 J and 100 J, according to STANAG 4489 [23]. The impact sensitivity of the composite HMX-AAc/EtAc was evaluated using the Kast fall hammer instrument. For this measurement, 40 μL of each sample was placed between two metallic cylinders fixed in a bigger metallic ring. The sensitivity to impact was evaluated as the energy (height multiplied by the gravity of the hammer) necessary to initiate 50% of the samples.

The electrostatic sensitivity test (ESD) [24] was utilized to establish the sensitivity of EMs to initiation by electrostatic discharge. This test simulates a potential scenario in which EMs spontaneously ignite during the production, processing, loading, or demilitarization stage. The testing of PBX was performed using an Electric Spark Tester—ESD 2010 EN instrument.

The vacuum stability test was performed following NATO Standard—STANAG 4556 [25], and it was used for evaluating the chemical stability and compatibility of energetic materials and for quality tests of energetic ingredients. The thermal vacuum stability test involves the artificial aging of the PBX at 100 °C for 40 h and measuring the pressure inside the test tube. Chemical stability was reported as the specific volume of gases released in this time interval.

The small-scale shock reactivity test (SSRT) [26,27,28], performed on an SSRT system (Figure S2 from the Supplementary Information File), measures the shock reactivity (explosiveness) of energetic materials, below critical diameter, without requiring a transition to detonation. The test setup combines the benefits of a lead block and gap tests. Compared to gap tests, the advantage is using a much smaller sample size of the tested explosive (approximately 500 mg). The sample volume *V_s_* is recommended to be 0.284 mL (284 mm^3^). The sample weight *m* was calculated using the formula *V_s_ × ρ ×* 0.95. The dent sizes were measured by filling them with powdered SiO_2_ and measuring the resulting weight.

The detonation velocity of the composite HMX-AAc/EtAc was determined using an oscilloscope PicoScope 3424 by measuring the time interval needed by the detonation wave to travel a known distance between two measuring probes placed at a known distance from each other. The detonation parameters were determined using the program EXPLO 5 [29,30]. EXPLO 5 is a thermochemical computer code that predicts the detonation (e.g., detonation velocity, pressure, energy, heat, temperature, etc.) and combustion (e.g., specific impulse, force, pressure, etc.) performance of energetic materials. The program uses the Becker–Kistiakowsky–Wilson (BKW) and Jacobs–Cowperthwaite–Zwisler (JCZ3) equation of state for gaseous detonation products, the ideal gas and virial equations of state of gaseous combustion products, and the Murnaghan equation of states for condensed products.

## 3. Results and Discussions

The AAc/EtAc copolymer binders, the EMs (RDX and HMX), and the resulting PBXs were characterized through specific analytical investigations, safety, and performance tests following NATO standards to comprehensively evaluate the performance and safety characteristics of the novel materials developed through this study.

The first investigation performed consisted of solubility tests to choose the proper binder for the following steps in PBX synthesis. Therefore, the solubility tests performed on the copolymers in alkaline solutions (after 24 h) showed that the 7:3 (AAc: EtAc) molar ratio copolymers display a good solubility after 24 h at pH higher than 11 and temperatures higher than 30 °C, so this binder was chosen for further development of PBX. The results are detailed in Table S1 from the Supplementary Information File.

The next step of this research involved the coating of the explosive crystals with the selected pH-sensitive copolymeric binder (7:3 molar ratio AAc:EtAc).

Two distinct manufacturing methods were utilized for the coating process: the first was the “classical” method—consisting of the dissolution of the copolymeric binder in DMF, wet mixing with the EM (HMX in this case), followed by sieve-granulation and solvent evaporation. The second was a “green” method consisting of the precipitation of the copolymeric binder on the surface of the explosive crystals based on the pH and temperature change.

The quantity of the polymeric solution was calculated to afford a final PBX composition consisting of approximately 10% binder and 90% high explosive.

Figure S1e,f from the Supplementary Information File illustrates the visual aspect of HMX soaked in DMF polymer lacquer and the coated HMX particles (PBX 3) after drying/granulation processes, respectively.

Figure S1a–d from the Supplementary Information File displays PBX 1 and PBX 2 formulations obtained via precipitation. Precipitation was performed for two concentrations of the acidic suspension of RDX. The PBX 1 mixture was obtained using RDX suspensions in 0.1 N HCl solutions, while PBX 2 formulation was obtained using RDX suspensions in 0.5 N HCl. In the case of PBX 1, it can be observed that after drying (Figure S1b), there are agglomerations (clumps) of particles exhibiting a lack of homogeneity, which may pose problems for further processing of the composition (batching and pressing) if the polymer is more abundant in the clumps. On the other hand, it can be noticed that for the PBX 2 composition (Figure S1d), the aggregation is reduced (only sporadic and relatively small clumps can be observed), and the homogeneity of the granules gives good perspective for further processing, for the final granular mixture PBX 2. Therefore, PBX 2 appeared suitable for producing explosive charges using pressing procedures, and no organic solvent was utilized in the manufacturing process. Consequently, we can affirm that employing a pH-sensitive binder for precipitation on the explosive crystals can be considered an “eco-friendly” method.

**Figure 2:**
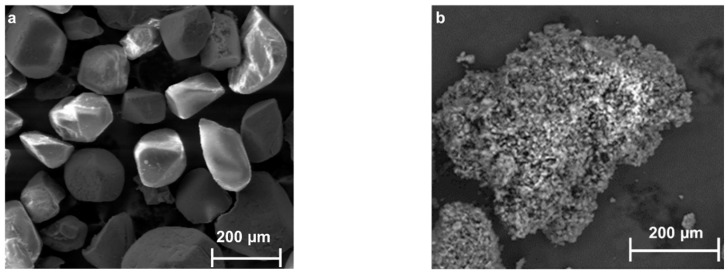

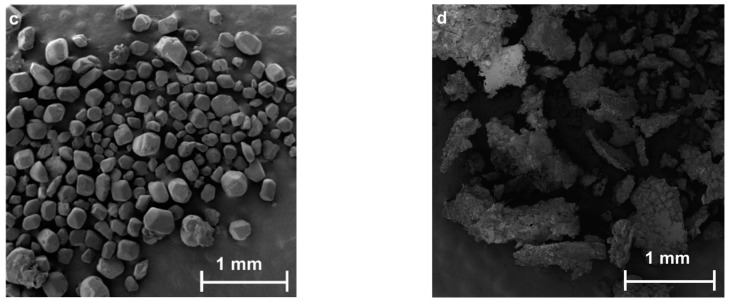
SEM images of (**a**)—HMX and (**c**)—RDX crystals and PBX compositions (**b**—PBX 3, **d**—PBX 1).

The SEM images displaying the morphology of the EMs utilized in this study (HMX and RDX) and of the resulting PBXs formulations are presented in Figure 2.

Figure 2d shows that the surface of the RDX crystals is fully covered with a polymeric amorphous layer, indicating that the polymer adhered well to the RDX crystals during precipitation. In Figure 2b, it can be observed that the binder has homogeneously covered the HMX particles. The white spots in SEM images appear probably due to the electrostatic charge of EMs.

**Figure 3:**
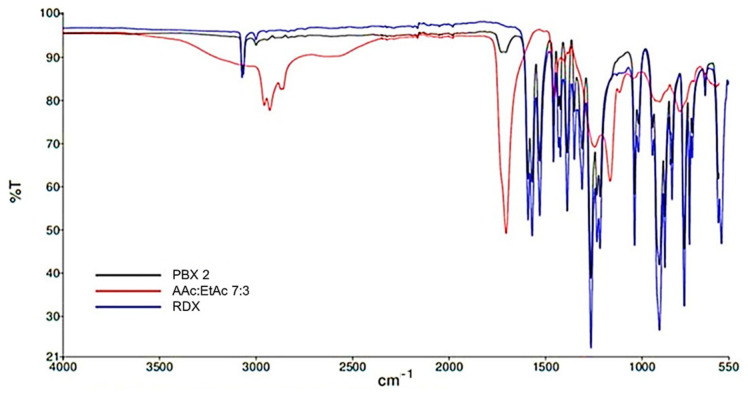
FT-IR spectra for the RDX, copolymeric binder, and PBX 2.

The FT-IR multigraph of the PBX compositions (Figure 3) shows the specific vibrations of both the RDX (especially those corresponding to the antisymmetric stretching vibration of the nitro groups at 1600 cm^−1^, symmetric stretching vibration of nitro groups at 1272 cm^−1^) and AAc/EtAc copolymer. Due to the low polymer concentration, the most visible signal is the absorption of the carboxyl group at 1732 cm^−1^. For the same reason, low binder concentration, but also for ease of readability, the PBX 3 and HMX spectra were not introduced in this FT-IR multigraph. However, HMX exhibits the same strong, broad absorption feature near 1550 cm^−1^, most likely associated with NO_2_ asymmetric stretch. As for RDX, the NO_2_ symmetric stretch in HMX is visible at around 1287 cm^−1^. A strong doublet near 950 cm^−1^ may be related to the N-O stretch, which has a reported range of 950 to 850 cm^−1^ in energetic materials, even though some studies attribute vibrations in the spectrum of HMX in this region to the N-N-C modes [31,32].

Differential thermal analysis (DTA) has been routinely used to evaluate materials capable of thermal autoignition [33] due to the accuracy of revealing the difference in temperature between the specimen and the reference for a given heat input. The EMs (RDX and HMX), the copolymeric binders, and the resulting PBX were subjected to DTA analysis to investigate the transitions that occur with the temperature increase. Table 2 summarizes the thermal properties of the investigated materials.

The thermograms of PBX 1 and PBX 2 (Figure S3 from the Supplementary Information File) display a sharp endothermic peak at 205 °C and 204 °C, respectively, corresponding to the melting point of the energetic composites. The endothermic peak attributed to the neat RDX melting was observed at 205 °C. Moreover, the DTA results indicate that the presence of the polymer has almost no influence on the temperature sensitivity of the RDX, the onset temperature for the decomposition of the PBX 1 and PBX 2 compositions being 207–208 °C, which is very close to the value of 209 °C, which is well-known from the literature as the onset temperature for the decomposition of RDX type 1 [34]. The DTA analysis revealed that the melting temperature of the energetic composite PBX 3 (HMX-AAc/EtAc) started at around 120 °C and ended at 180 °C. Thus, the polymeric binder decreases the melting temperature of PBX 3, compared to the melting temperature of the raw HMX, which is approximately 190 °C [35]. The thermal ignition temperature of the new composite PBX 3 (HMX-AAc/EtAc) was around 260 °C. The presence of the polymer leads to a decrease in the deflagration point, which is expected given that for nitramine, the decomposition temperature is related to the melting temperature, which is determined by impurities in the mixture. The decomposition of HMX reaches the maximum at 285 °C [35]. The difference between the two values, the decomposition temperatures of the new composite and the raw HMX, is only 20 °C, concluding that the temperature sensitivity of the new composite HMX-AAc/EtAc is still low.

The next section of the experimental study involved the evaluation of the safety characteristics of the novel PBXs, following the NATO standards described in Section 2.2. To obtain a general estimation of the polymer coating’s influence on the sensitivity of the RDX or HMX, friction sensitivity tests were performed using a BAM apparatus and several loading forces. The results, presented in Table 3 and Table 4, indicate that for both PBX 1 and PBX 2, the friction sensitivity is reduced dramatically (the loads for the 50% reaction were higher than 288 N, compared to 120 N for pure RDX) [36]. The friction sensitivity of PBX 3 was determined on the same BAM Friction Tester. The tests started at 100 N, the friction sensitivity of HMX raw material [10]. No ignitions were observed, not even with the maximum load corresponding to 360 N. In conclusion, the new composite HMX-AAc/EtAc is insensitive to this stimulus.

Since HMX and, subsequently, PBX 3 are more sensitive than RDX-based PBXs at mechanical stimulus, the following investigations, which imply high risks for the operator, were performed only for the HMX-based composite.

The impact sensitivity test provides information about the minimum energy required to initiate the decomposition of an energetic material. The hammer was released over the samples from different heights, and the energies were calculated based on the mass. The impact sensitivity of the new composite PBX 3 (HMX-AAc/EtAc) was 12.5 J. Thus, PBX 3 is less sensitive than the raw material HMX, which has an impact sensitivity of 5.2 J [37].

Electrostatic discharge is one of the most frequent and the least characterized causes of accidental explosions of energetic materials. Reliable data on the electrostatic spark sensitivity of energetic materials is critical in manufacturing, quality control, explosives processing, loading, transportation, storage, demilitarization, and research and development of new explosive materials. The electrostatic discharge sensitivity of the new composite HMX-AAc/EtAc was determined to be 0.7 J (Table 5), which classifies the composition as less sensitive to electrostatic discharge (energy value between 0.5 and 5 J) compared to 0.170 J for pure HMX (classified as sensitive to electrostatic discharge, the energy was between 0.05 and 0.5 J), as presented in the literature [38].

The thermal stability of the PBX 3 composite was evaluated by measuring the volume of gaseous reaction products generated when the samples were kept at 100 °C for 40 h to simulate the aging process of the energetic material. This is a good sign of the chemical compatibility between the explosive filler and the binder. The energetic materials with a specific volume greater than 2 cm^3^/g are considered to have reduced chemical stability [39]; therefore, the new composite HMX-AAc/EtAc can be regarded as chemically stable since the specific volume obtained was 0.352 cm^3^/g.

The SSRT test setup, used to evaluate the shock sensitivity, is presented in Figure S2 from the Supplementary Information File. The tests were performed by comparison with two consecrated insensitive explosives 1,1-diamino-2,2-dinitro ethylene (FOX 7) and 3-nitro-1,2,4-triazol-5-one (NTO). The results obtained for the SSRT test are shown in Table 6 below.

Table 6 shows that the binder significantly reduces the shock sensitivity; thus, for the new compound HMX-AAc/EtAc, the shock sensitivity is comparable with FOX-7 and slightly higher than nitrotriazolone shock sensitivity (NTO). 

For the following investigations, the binder percentage was varied to evaluate its influence on the detonation performances of the PBXs. The detonation velocities obtained for different binder percentages in PBX 3 composites are shown in Table 7. The detonation parameters of the new HMX-AAc/EtAc composites were assessed using the program EXPLO-5 and are summarized in Table 8. The program is designed so that it enables the calculation of chemical equilibrium composition and thermodynamic parameters of the state along the adiabat of shock detonation products, the C-J state, and the detonation parameters at the C-J state, as well as the parameters of the state along the expansion isentrope. It can be observed that the detonation rate values decrease with the increase of binder concentration in the explosive mixture. The detonation velocities values obtained by EXPLO5 simulation are 3.5 % higher than the ones obtained by experimental tests but still in good accordance with the experimental data.

The last stage of this study evaluated the possibility of recovering the explosive crystals from the novel PBX formulations and investigating the recovered material’s characteristics. Following the procedure described in Section 2.2 (iv), the energetic material, HMX, was recovered and analyzed. Table 9 shows the HMX recovery yield. The tests were performed only on the HMX-based PBX due to the higher chemical stability of HMX in basic aqueous solutions.

The recovered material is a white and odorless compound. Due to their size, a portion of the smaller octogen particles (<50 μm) were lost during filtration, resulting in an average recovery value of 89%.

The recovered HMX was investigated by various characterization methods: infrared spectroscopy (FT-IR), differential thermal analysis (DTA) (see Supplementary Figure S3), impact sensitivity test (BAM Fall Hammer Test), friction sensitivity test (BAM Friction Test), vacuum stability test, and scanning electron microscopy. FT-IR plot obtained for the recovered material was compared with pure HMX spectra, and the characteristic peaks were found to be consistent with the literature data. All the results obtained following the stability and sensitivity tests (DTA, vacuum stability test, impact sensitivity test, and friction sensitivity test) agreed with the specific data for raw HMX.

**Figure 4:**
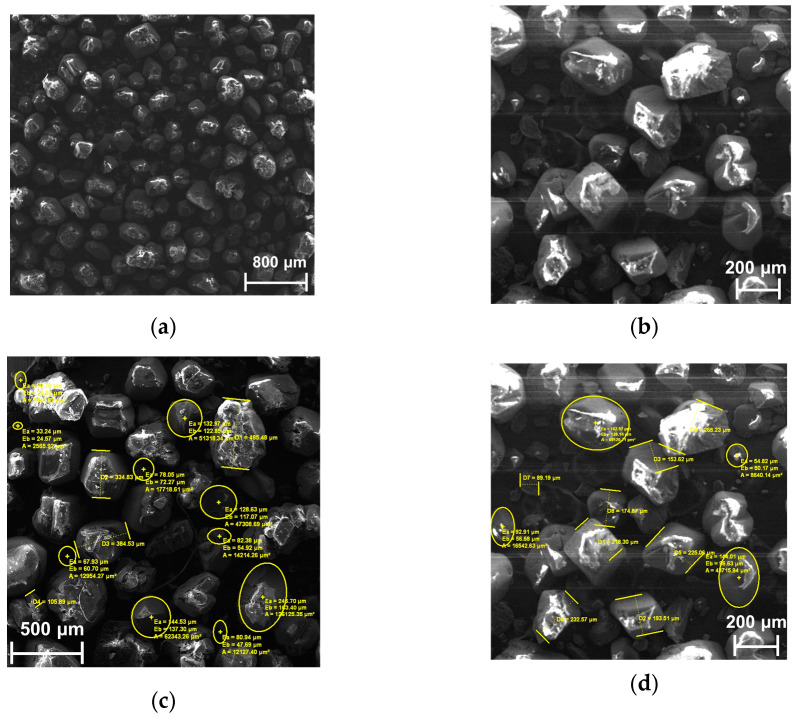
(**a**) HMX used to obtain the new composite HMX-AAc/EtAc; (**b**) HMX recovered from PBX 3; (**c**) raw HMX particle size; (**d**) recovered HMX particle size.

The SEM images presented in Figure 4 show that no significant morphology changes of HMX crystals occur during the removal of the AAc/EtAc copolymer binder. A graph indicating the particle size distribution extrapolated from SEM analysis is also presented in the Supplementary Material (Figure S4). A slight decrease in particle size could be ascertained, which, together with the technological loss in filtration and manipulation operations in rather small batches, can explain the HMX recovery yield being less than the total.

## 4. Conclusions

This study presents the design and characterization of a “green” coating technique to obtain a polymer coating on the surface of RDX and HMX crystals that enables a simple, safer method to produce PBXs and recover the explosive material without the use of organic solvents. A laboratory-scale solvent-free technology was demonstrated for the manufacture of RDX-based PBX compositions using a prior developed acrylic acid–ethyl acrylate (AAc/EtAc) copolymer binder system with adjustable water solubility at alkaline pH. A rapid change of the pH and temperature leads to the precipitation of the polymer from an alkaline aqueous solution on the surface of the RDX crystals suspended in an acidic aqueous solution. The best results for specific addition and stirring rates were obtained in 0.5 N HCl solutions when homogenous, non-adherent granules of PBX were obtained, giving a good perspective for further development of pressed explosive charges without using any organic solvent. The SEM analysis confirmed good dispersion of the polymeric layer onto the surface of the RDX crystals. The presence of the polymer in the composition was confirmed by FT-IR analysis. Estimative sensitivity tests showed a significant decrease in friction sensitivity (compared to RDX) and no influence on temperature sensitivity.

This study also aimed to obtain a new energetic composite based on HMX and a polymeric binder (based on acrylic acid and ethyl acrylate) soluble in water at a slightly alkaline pH. For the production and use of the new explosive mixture in different applications, it was necessary to characterize the physical, chemical, and explosive properties.

To evaluate the explosive safety properties, the HMX-AAc/EtAc composite (PBX 3) was subjected to specific analyses to determine the chemical stability in vacuum (STANAG 4556), temperature sensitivity (STANAG 4491), impact sensitivity (STANAG 4489), sensitivity to friction (STANAG 4487), and sensitivity to electrostatic discharge (STANAG 4490). The explosive performance characteristics of the HMX-AAc/EtAc high explosive mixture were determined experimentally by determining the detonation velocity. Using the EXPLO5 program and the Jacobs–Cowperhwaite–Zwisler (JCZ3) and Becker–Kistiakowsky–Wilson (BKW) methods, the detonation parameters of the HMX-AAc/EtAc explosive mixture were determined for different binder ratios (5%, 10%, and 20%). All the results obtained were in concordance with specific literature data.

Using a solvent granulation process and then a hydraulic press, six cylindrical charges were obtained for HMX-AAc/EtAc composites containing 2 g of HMX. An alkaline solution (pH = 11) was used to recover the energetic material from the cylindrical PBX 3 composite charges. An HMX average recovery yield of 89% was obtained.

The recovered energetic material was characterized by infrared spectroscopy (FT-IR), differential thermal analysis (DTA), the impact sensitivity test (BAM Fall Hammer Test), friction sensitivity test (BAM Friction Test), vacuum stability test, and scanning electron microscopy. All the results obtained agree with the literature data for raw HMX.

## Figures and Tables

**Table 1:** Composition of energetic composites.

Sample Code	Binder ^‡^ [g]	RDX [g]	HMX [g]	DMF * [mL]	C_HCl_ ** (N)	C_NaOH_ *** (N)
PBX 1	1	9	-	-	0.1 N	0.1 N
PBX 2	1	9	-	-	0.5 N	0.1 N
PBX 3	1	-	9	16	-	-

* solvent for binder coating in PBX 3; ** normal concentration of HCl solution employed for RDX crystals dispersion; *** normal concentration of alkaline solution for binder dissolution; ^‡^ molar ratio between monomers AAc/EtAc 7:3.

**Table 2:** Thermal properties of energetic composites.

Sample Code	Melting Onset [°C]	Melting End [°C]	Decomposition Onset [°C]	Decomposition Maximum [°C]	Decomposition End [°C]
PBX 1	200	208	209	210	216
PBX 2	198	207	208	211	219
PBX 3	120	180	260	265	270
RDX	202 *		209 *		
HMX	190 *		285 *		

* reference values from the literature.

**Table 3:** Friction sensitivity test results for PBX 1 (R—reaction).

	1	2	3	4	5	6	7	8	9	10
F1 (288 N)	R	X	X	R	X	X	R	R	X	X
F2 (324 N)	R	X	R	X	R	R	R	X	R	R
F3 (360 N)	R	R	R	R	R	R	R	R	R	R

**Table 4:** Friction sensitivity test results for PBX 2 (R—reaction).

	1	2	3	4	5	6	7	8	9	10
F1 (288 N)	X	X	X	X	X	X	X	X	X	X
F2 (324 N)	X	X	X	R	R	R	R	R	X	X
F3 (360 N)	R	R	R	R	R	X	X	R	R	R

**Table 5:** Comparative summary of sensitivity tests.

Sample Code	Temperature Sensitivity [°C]	Friction Sensitivity [N]	Impact Sensitivity [J]	Electrostatic Discharge Sensitivity [J]
PBX 1	209	>288	-	-
PBX 2	208	324	-	-
PBX 3	260	>360	12.5	0.7
RDX	209 *	120 *	-	**-**
HMX	285 *	100 *	5.2 *	0.17 *

* reference values from the literature.

**Table 6:** SSRT test results.

Sample	Sample’s Mass [mg]	SiO_2_ Mass [mg]
NTO	520	455
PBX 3	432	568
FOX-7	503	579

**Table 7:** Detonation velocities.

HMX-Aac/EtAc, Binder [%]	Density [g/cm^3^]	Time [μs]	Detonation Velocity [m/s]
5% binder	1.85	11.67 ± 0.05	8569 ± 39
10% binder	1.82	11.85 ± 0.05	8439 ± 39
20% binder	1.76	12.38 ± 0.05	8078 ± 39

**Table 8:** Detonation parameters (JCZ method).

Detonation Parameters	HMX [%]—Aac/EtAc [%]						
	100-0 (1.96 g/cm^3^)	100-0 (1.90 g/cm^3^)	100-0 (1.85 g/cm^3^)	95-5	90-10	85-15	80-20
Heat of detonation [kJ/kg]	5695.18	5692.39	5683.23	5325.89	5161.73	4986.09	4792.75
Detonation temperature [K]	3555.55	3608.01	3658.52	3242.65	3146.67	3038.521	2916.38
Detonation pressure [Gpa]	40.70	38.15	35.78	35.61	30.81	26.54	22.89
Detonation velocity [m/s]	9497	9267.15	9044	8910.65	8622.01	8329.88	8028.68

**Table 9:** HMX recovered from the PBX 3.

Temperature [°C]	Cylinder Charge Mass [g]	Recovered Mass [g]	Recovered Mass [%]
20	2	1.82	91
20	2	1.78	89
20	2	1.80	90
30	2	1.86	93
30	2	1.69	85
30	2	1.73	87
			**89**

## Data Availability

The data presented in this study are available on request from the corresponding author.

## Supplementary Materials

The following supplementary information can be downloaded at: https://www.mdpi.com/article/10.3390/polym15071790/s1. The following supplementary materials are included: Table S1: Solubility tests on AAc/EtAc copolymers in alkaline solutions after 24 h; Figure S1: Images of the energetic composites obtained—(a), (b)—PBX 1; (c,d)—PBX 2; (e,f)—PBX 3; (a,c,e)—wet precipitate; (b,d,f)—dry particles; Figure S2: SSRT test setup; Figure S3: DTA thermograms for RDX, PBX 1, and PBX 2 composite; Figure S4: Dimensional distribution of the HMX crystals. Reference [40] is cited in the supplementary materials.

## References

[1] Anand S., Celin S.M., De Luca L.T., Shimada T., Sinditskii V.P., Calabro M. (2017). Green Technologies for the Safe Disposal of Energetic Materials in the Environment. Chemical Rocket Propulsion: A Comprehensive Survey of Energetic Materials.

[2] Klapötke T.M., Scharf R., Stierstorfer J., Unger C.C. (2021). Toxicity Assessment of Energetic Materials by Using the Luminescent Bacteria Inhibition Test. Propellants Explos. Pyrotech..

[3] Bondarchuk S.V. (2023). Prediction of aquatic toxicity of energetic materials using genetic function approximation. FirePhysChem.

[4] Wang Q., Xu L.-P., Deng C.-Q., Yao E.-G., Chang H., Pang W.-Q. (2023). Characterization of Electrospinning Prepared Nitrocellulose (NC)-Ammonium Dinitramide (ADN)-Based Composite Fibers. Nanomaterials.

[5] (2001). Safe Disposal of Munitions, Design Principles and Requirements, and Safety Assessment..

[6] Sarah M., Stott N. (2003). Destroying Surplus Weapons.

[7] Voicu A.E., Rotariu T., Teodorescu M., Zecheru T., Tiganescu T.V., Orban O. (2017). pH Sensitive Polymeric Binders for Energetic Materials. Mater. Plast..

[8] Cumming A.S., Johnson M.S. (2019). Energetic Materials and Munitions: Life Cycle Management, Environmental Impact, and Demilitarization.

[9] Bar’yakhtar V.G., Rosendorfer T. (2012). Demilitarisation of Munitions: Reuse and Recycling Concepts for Conventional Munitions and Rocket Propellants.

[10] Chen P., Huang F., Ding Y. (2007). Microstructure, deformation and failure of polymer bonded explosives. J. Mater. Sci..

[11] Zalewski K., Chyłek Z., Trzciński W.A. (2021). A Review of Polysiloxanes in Terms of Their Application in Explosives. Polymers.

[12] Wilkinson J., Watt D. (2006). Review of Demilitarisation and Disposal Techniques for Munitions and Related Materials.

[13] Kim D., Kim H., Huh E., Park S., Lee C.-H., Ahn I.-S., Koo K.-K., Lee K.D. (2019). Effect of a polymer binder on the extraction and crystallization-based recovery of HMX from polymer-bonded explosives. J. Ind. Eng. Chem..

[14] Mitchell A.R., Coburn M.D., Schmidt R.D., Pagoria P.F., Lee G.S. (2002). Advances in the chemical conversion of surplus energetic materials to higher value products. Thermochim. Acta.

[15] Kang H., Kim H., Lee C.-H., Ahn I.-S., Lee K.D. (2017). Extraction-based recovery of RDX from obsolete Composition B. J. Ind. Eng. Chem..

[16] Talawar M.B., Sivabalan R., Mukundan T., Muthurajan H., Sikder A.K., Gandhe B.R., Rao A.S. (2009). Environmentally compatible next generation green energetic materials (GEMs). J. Hazard. Mater..

[17] Yang Z., Ding L., Wu P., Liu Y., Nie F., Huang F. (2015). Fabrication of RDX, HMX and CL-20 based microcapsules via in situ polymerization of melamine–formaldehyde resins with reduced sensitivity. Chem. Eng. J..

[18] Merline D.J., Vukusic S., Abdala A.A. (2013). Melamine formaldehyde: Curing studies and reaction mechanism. Polym. J..

[19] Kosareva E.K., Zharkov M.N., Meerov D.B., Gainutdinov R.V., Fomenkov I.V., Zlotin S.G., Pivkina A.N., Kuchurov I.V., Muravyev N.V. (2022). HMX surface modification with polymers via sc-CO_2_ antisolvent process: A way to safe and easy-to-handle energetic materials. Chem. Eng. J..

[20] Rotariu T., Enache C., Goga D.A., Toader G., Stancu I.C., Serafim A., Esanu S., Trana E. (2016). Theoretical and experimental studies on new plastic pyrotechnic compositions. Mater. Plast..

[21] Duan B., Li J., Mo H., Lu X., Xu M., Wang B., Liu N. (2021). The Art of Framework Construction: Core–Shell Structured Micro-Energetic Materials. Molecules.

[22] (2002). Explosive, Friction Sensitivity Tests.

[23] (1999). Explosives, Impact Sensitivity Tests.

[24] (2001). Standardization Agreement (STANAG) on Explosives, Electrostatic Discharge Sensitivity Tests..

[25] (1998). Explosives: Vacuum Stability Test.

[26] Sandusky H.W., Granholm R.H., Bohl D.G. (2010). Small-Scale Shock Reactivity and Internal Blast Test. U.S. Patent.

[27] Granholm R., Sandusky H. (2006). Small-Scale Shock Reactivity and Internal Blast Test. AIP Conf. Proc..

[28] Fischer N., Fischer D., Klapötke T.M., Piercey D.G., Stierstorfer J. (2012). Pushing the limits of energetic materials–the synthesis and characterization of dihydroxylammonium 5,5′-bistetrazole-1,1′-diolate. J. Mater. Chem..

[29] Sućeska M. (1999). Evaluation of detonation energy from EXPLO5 computer code results. Propellants Explos. Pyrotech..

[30] Klapötke T.M., Stierstorfer J. (2008). The New Energetic Compounds 1,5-Diaminotetrazolium and 5-Amino-1-methyltetrazolium Dinitramide–Synthesis, Characterization and Testing. Eur. J. Inorg. Chem..

[31] Brand H.V., Rabie R.L., Funk D.J., Diaz-Acosta I., Pulay P., Lippert T.K. (2002). Theoretical and Experimental Study of the Vibrational Spectra of the α, β, and δ Phases of Octahydro-1,3,5,7-tetranitro-1,3,5,7-tetrazocine (HMX). J. Phys. Chem. B.

[32] McNesby K.L., Pesce-Rodriguez R.A. (2002). Applications of Vibrational Spectroscopy in the Study of Explosives.

[33] Bohon R.L. (1961). Differential Thermal Analysis of Explosives and Propellants under Controlled Atmospheres. Anal. Chem..

[34] Pervukhin V.V., Sheven D.G. (2021). Acceleration of the thermal decomposition of RDX in microdroplets investigated by aerodynamic thermal breakup droplet ionization mass spectrometry. Aerosol Sci. Technol..

[35] Quintana J.R., Ciller J.A., Serna F.J. (1992). Thermal Behaviour of HMX/RDX Mixtures. Propellants Explos. Pyrotech..

[36] Köhler J., Meyer R., Homburg A. (2008). Explosives.

[37] Meyer R., Köhler J., Homburg A. (2016). Explosives.

[38] Pouretedal H.R., Damiri S., Sharifi A. (2020). Study of triplet kinetic of thermal decomposition reaction and sensitivity to impact and electrostatic discharge of HMX polymorphs. J. Therm. Anal. Calorim..

[39] Rotariu T., Pulpea B.-G., Dîrloman F.-M., Diacon A., Rusen E., Toader G., Zvîncu N.-D., Iordache T.-V., Botiș R.H. (2022). The Influence of Potassium Salts Phase Stabilizers and Binder Matrix on the Properties of Novel Composite Rocket Propellants Based on Ammonium Nitrate. Materials.

[40] Mayer R., Köhler J., Homburg A. (2002). Explosives.

