# Supplementary material for: “Green” PBX Formulations Based on High Explosives (RDX and HMX) and Water-Soluble pH-Sensitive Polymeric Binders
Source: Polymers (Basel). 2023 Apr 4;15(7):1790. doi: 10.3390/polym15071790 (PMC10098890; doi:10.3390/polym15071790)
Supplement: Supplementary file 1 [file polymers-15-01790-s001.zip › polymers-2289252-supplementary.pdf]

polymers

MDPI

*Supplementary information*

# "Green" PBX Formulations Based on High Explosives (RDX and HMX) and Water-Soluble pH-Sensitive Polymeric Binders

**Traian Rotariu [1], Andreea Elena Moldovan [1,*], Gabriela Toader [1], Aurel Diacon [1,2,*], Edina Rusen [2], Raluca Elena Ginghina [3], Ovidiu Iorga [3], Horia Răzvan Botiș [4] and Thomas Klapötke [5]**

[1] Military Technical Academy "Ferdinand I", 39–49 George Cosbuc Boulevard, 050141 Bucharest, Romania
[2] Faculty of Chemical Engineering and Biotechnologies, University Politehnica of Bucharest, 1–7 Gh. Polizu Street, 011061 Bucharest, Romania
[3] Research and Innovation Center for CBRN Defense and Ecology, 225 Olteniței Ave., 041327 Bucharest, Romania
[4] Compania Națională ROMARM S.A., 5 Timișoara Boulevard, 061301 Bucharest, Romania
[5] Department of Chemistry, Ludwig-Maximilian University of Munich, Butenandtstr. 5–13, 81377 Munich, Germany
* Correspondence: andreea.moldovan@mta.ro (A.E.M.); aurel_diacon@yahoo.com (A.D.)

**Table S1.** - Solubility tests on AAc/EtAc copolymers in alkaline solutions after 24 h.

| *NaOH solutions/ temperature* | AAc/EtAc | | | | | |
|---|---|---|---|---|---|---|
| | 5:5 | | | 7:3 | | |
| | 20 °C | 30 °C | 50 °C | 20 °C | 30 °C | 50 °C |
| pH 11 | - | - | - | - | + | + |
| pH 12 | - | - | + | + | + | + |
| pH 13 | + | + | + | + | + | + |

(-) polymers did not dissolve; (+) polymers dissolved.

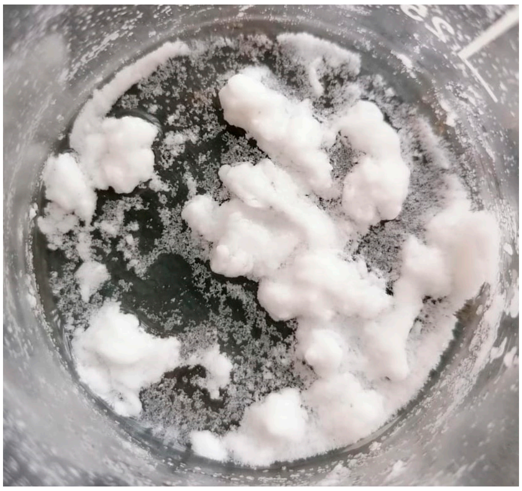

a

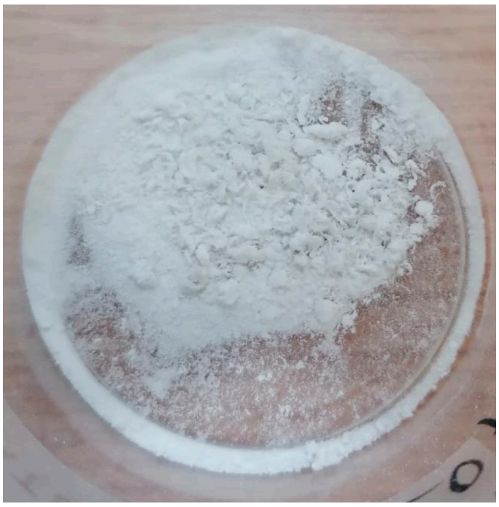

b

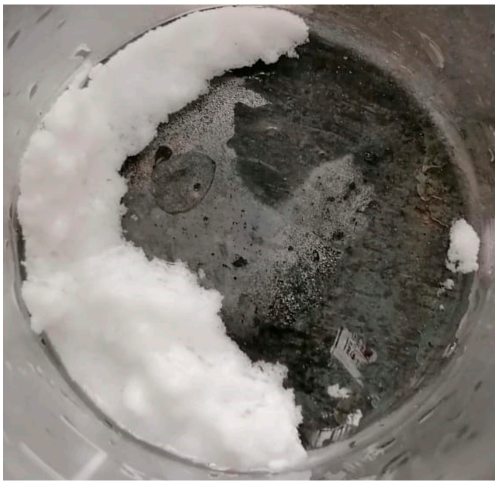

**c**

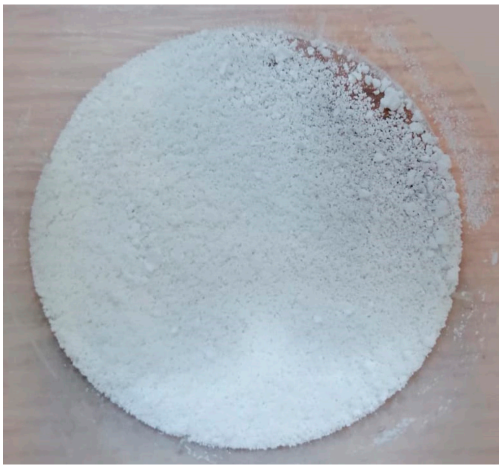

**d**

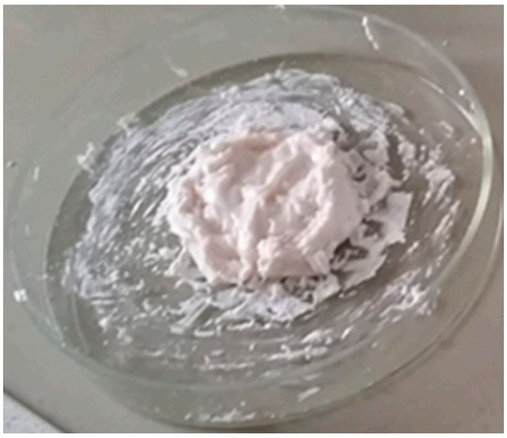

**e**

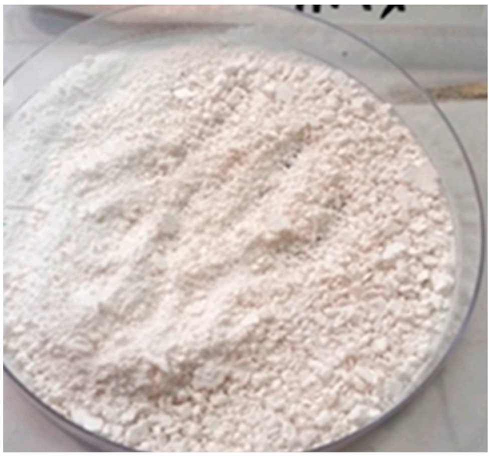

**f**

**Figure S1. –** Images of the energetic composites obtained. a, b – PBX 1; c, d – PBX 2; e, f – PBX 3; a, c, e – wet precipitate; b, d, f – dry particles

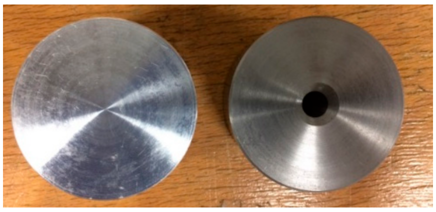

a) Aluminum block (left), steel block (right) mode

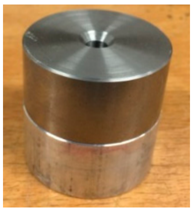

b) blocks positioning

The steel blocks are filled with the sample.

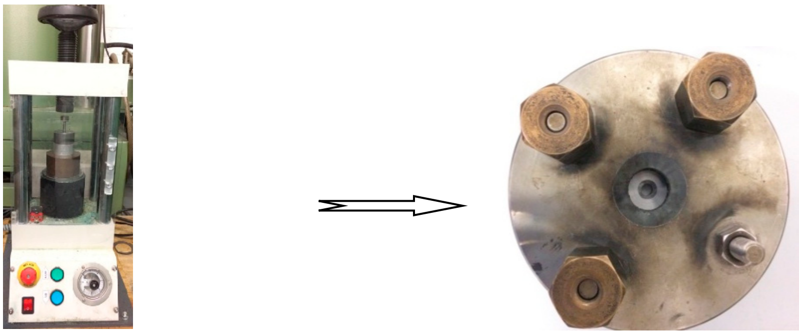

c) sample pressing

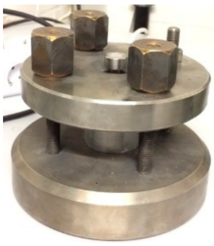

d) Device for fixing the blocks needed for the test

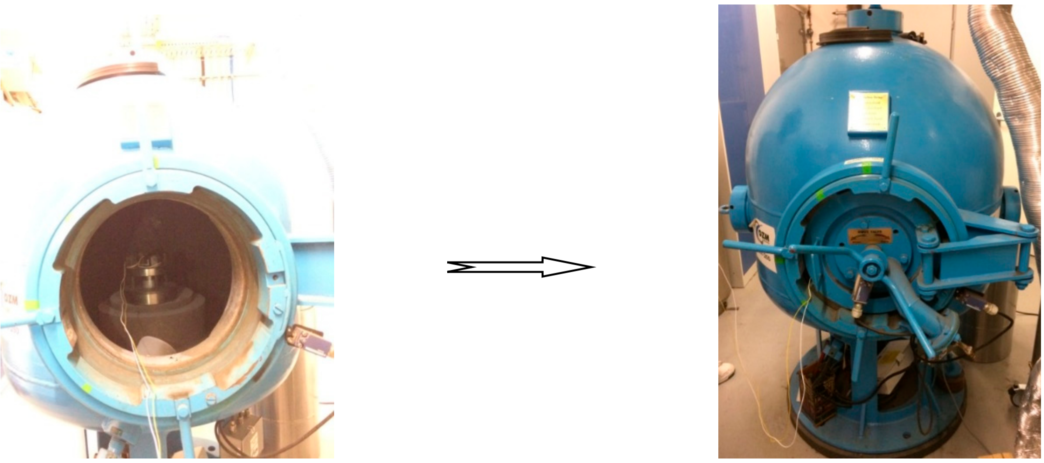

e) detonation chamber

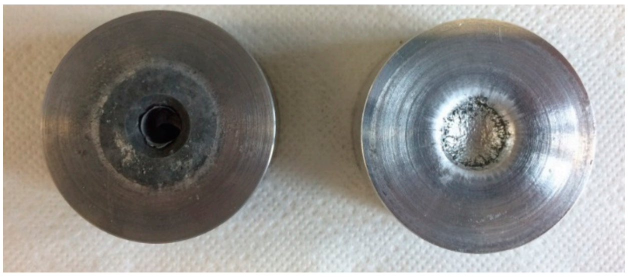

f) results after testing (left)- indentation filled with $SiO_2$ (right)

**Figure S2.** – SSRT test setup.

The SSRT measures the shock reactivity (explosiveness) of energetic materials, often well-below critical diameter, without requiring a transition to detonation. The test setup combines the benefits of the lead block test ([1] pp. 197-200) and the gap test ([1] p. 148). Compared to the gap test, the advantage is the use of a much smaller amount of sample (ca. 500mg). The sample volume (Vs) is recommended to be 0.284mL (284 $mm^3$). The mass of the sample was determined using the formula Vs x $\rho_{sample}$ x 0.95.

The two blocks are placed on top of each other (the aluminum block at the bottom Figure S2 a,b). The amount of sample resulting from the calculation is inserted into the hole of the steel block. The sample is manually pressed (Figure S2c)) and then a detonator is inserted into the same hole. The two blocks are fixed (Figure S2d)) and introduced in the detonation chamber (Figure S2e)). The dimensions of the indentations (Figure S2 f)) formed after the test were measured and filled with $SiO_2$, thus determining the resulting mass.

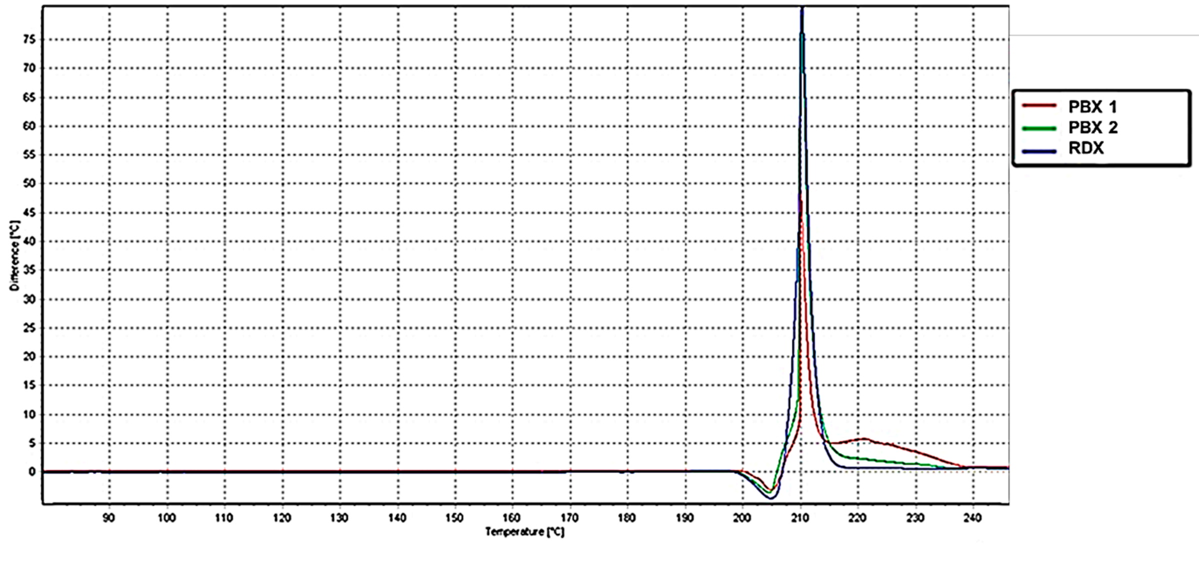


**Figure S3.** – DTA thermograms for RDX, PBX 1 and PBX 2 composite.

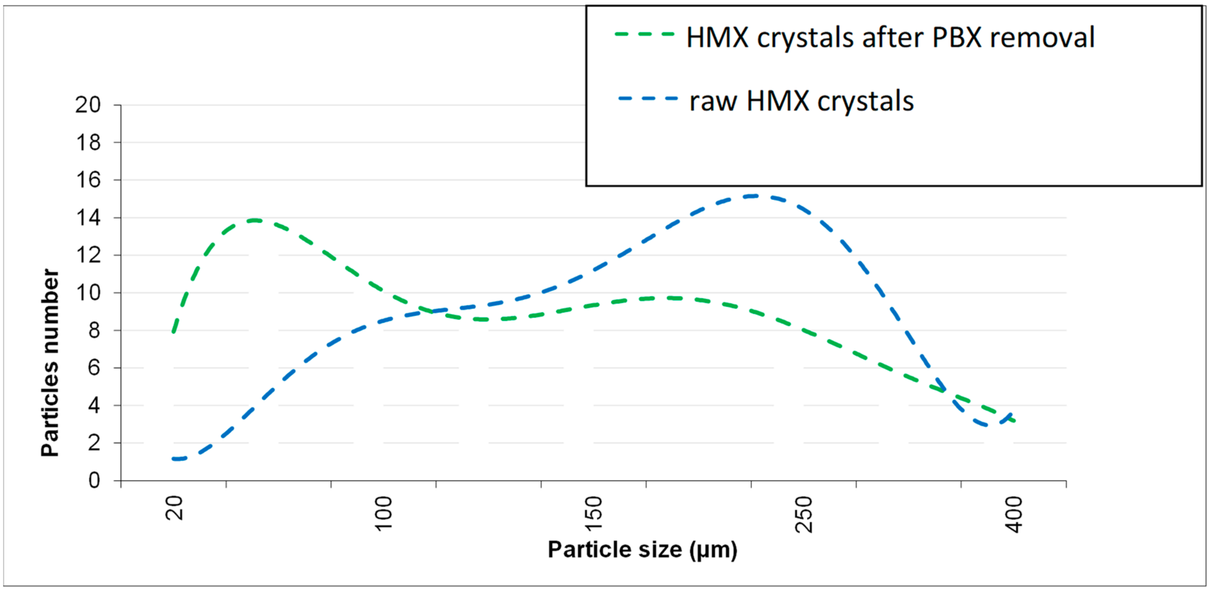


**Figure S4.** – Dimensional distribution of HMX crystals obtained from SEM images dimensional measurements.

**References:**

[1] Mayer, R.; Köhler, J.; Homburg, A., *Explosives*, 5th ed., Wiley VCH, Weinheim, **2002**, pp. 148, 197-200;
